# MiR1918 enhances tomato sensitivity to *Phytophthora infestans* infection

**DOI:** 10.1038/srep35858

**Published:** 2016-10-25

**Authors:** Yushi Luan, Jun Cui, Weichen Wang, Jun Meng

**Affiliations:** 1 School of Life science and Biotechnology, Dalian University of Technology, Dalian 116024, China; 2 School of Computer Science and Technology, Dalian University of Technology, Dalian 116024, China

## Abstract

Late blight of tomato is caused by the oomycete pathogen *Phytophthora infestans*. In our previous work, we identified and characterized a miR1918 in *P. infestans* (pi-miR1918), and showed that its sequence is similar to the sequence of tomato miR1918 (sly-miR1918). In this study, we used *Arabidopsis thaliana* pre-miR159a as a backbone to synthesize pi-miR1918 via PCR and mutagenesis. The artificial pi-miR1918 was used to investigate the role of miR1918 in tomato-*P. infestans* interaction. Trangenic tomato plants that overexpressed the artificial pi-miR1918 displayed more serious disease symptoms than wild-type tomato plants after infection with *P. infestans*, as shown by increased number of necrotic cells, lesion sizes and number of sporangia per leaf. The target genes of pi-miR1918 and sly-miR1918 were also predicted for tomato and *P. infestans*, respectively. qPCR analysis of these targets also performed during tomato-*P. infestans* interaction. The expression of target gene, RING finger were negatively correlated with miR1918 in the all Lines of transgenic tomato plants. In addition, we used the 5′ RACE to determine the cleavage site of miR1918 to RING finger. These results suggested that miR1918 might be involved in the silencing of target genes, thereby enhancing the susceptibility of tomato to *P. infestans* infection.

In the natural environment, plants usually coexist with a diversity of microorganisms. These microorganisms also include a wide range of harmful pathogens such as viruses, bacteria, fungi and oomycetes, which may present a continuous threat to the plants^1^. Some of these pathogens have acquired strategies that would facilitate their infection of the plants, and in response to this, the plants have also developed their own defenses against such infection. During the infection, the pathogens produce effectors to increase their pathogenicity. However, plants can sense the pathogens using an innate immune system that is based on miR1918 PAMP-triggered immunity (PTI) and effector-triggered immunity (ETI)^2^,^3^.

Plant small RNAs (sRNAs), including microRNAs (miRNAs) and small interfering RNAs (siRNAs), are important regulatory effector**s** in plant-pathogen interaction. They regulate their targets by sRNA-mediated gene silencing pathways. In a recent study, the expression levels of cotton miR482s were found to be down-regulated and several nucleotide-binding sites containing leucine-rich repeat (NBS-LRR) targets were found to be up-regulated upon infection by the fungal pathogen *Verticillunm dahiae*^4^. Most plant disease resistance genes (R gene) belong to the NBS-LRR gene family, which plays important roles in ETI^5^. In microbe-inoculated tobacco, the levels of NBS-LRR proteins were found to increase as a result of reduced miR6019 and miR6020-mediated suppression of NBS-LRR^6^. In addition, the expressions of plant miRNAs, such as miR160, miR167 and miR393 can be induced, which may contribute to PTI during pathogen attacks^7^,^8^,^9^. Beside the R genes, various plant transcription factors also play an important role during plant-pathogen interaction. For example, the tae-miR1136-p3 in wheat was found to be down-regulated when wheat was inoculated with stripe rust, displaying 2- and 1.5-fold reductions in expression at 48 and 120 h post inoculation (hpi), respectively, compared to control wheat, while the expression of its target gene (a zinc finger) was found to be highly induced at 48 hpi and 120 hpi, yielding 2.34- and 8.50- fold increases, respectively^10^. Unlike plant miRNAs, which have been extensively studied, the study of pathogen miRNAs and their roles as important regulators of gene expression is very limited. A number of miRNA have been identified in the plant pathogenic fungus *Sclerotinia sclerotiorum* by high-throughput sequencing. The functions of 2 milRNAs and 42 milRNA candidates have been studied by sequence analysis, northern blot and RT-PCR^11^.

Small RNAs (sRNAs) have been reported to move horizontally between plant and pathogen^12^. A total of 37 tomato miRNA and 38 tomato miRNA* sequences have been predicted, and most of the miRNA and miRNA* have high complementarity with the open reading frames (ORFs) of the genomic RNAs of CMV-Fny and CMV-Q, respectively, suggesting that these miRNAs may repress the translation or induce the cleavage of target genes^13^. In addition to plant miRNAs that can be transferred into viruses, several pathogens have also been shown to use RNA interference to suppress the expression of host genes^14^,^15^. For instance, genome-wide identification has predicted that vsiRNAs from Grapevine fleck virus (GFkV) and Grapevine rupestris stem pitting associated virus (GRSPaV) may target plant transcripts^16^. Three *Botrytis cinerea* sRNAs (Bc-SiR3.1, Bc-siR3.2 and Bc-siR5) have been shown to target different sets of *A. thaliana* genes involved in immunity against *B. cinera*^10^,^17^. Bc-siR3.1 targets the oxidative stress-related gene *peroxiredoxin,* whereas Bc-siR3.2 targets the *mitogen-activated protein kinase 1 and protein kinase 2* gene (MPK1 and MPK2), while Bc-siR5 targets the *cell wall-associated kinase* gene.

Artificial miRNA (amiRNA) has emerged as a new gene regulation strategy, designed to suppress pathogen genes. AmiRNA was engineered by replacing the mature miRNA/miRNA* sequence with the complementary sequence taken from the host miRNA precursors^18^,^19^,^20^. At present, the precursors of *Arabidopsis* miR319, miR159, miR168 and miR171 are being used as the backbones to construct amiRNAs. For instance, two amiRNAs have been constructed from the precursor of *Arabidopsis* miR159; one targets the overlapping sequence between CMV2a and 2b, and the other targets the untranslated region (UTR) of cucumber mosaic virus (CMV). Transgenic tomato plants overexpressing these two amiRNAs develop good resistance against CMV^21^. In addition, transient expression of vsiRNA-4A as amiRNAs in *Nicotiana benthamiana* can cause leaf twisting and stunting^22^. Overall, amiRNA provides a new way to study the function of sRNAs.

Tomato (*Solanum lycopersicum*) is a major crop plant that is widely cultured in the world^23^. However, field-grown tomatoes are susceptible to disease such as late blight (LB), which is one of the most devastating diseases of field-grown tomatoes, and it can cause severe economic losses for tomato growers^24^,^25^,^26^. Late blight is caused by the pathogen *Phytophthora infestans*. Our previous work have focused on the use of high-throughput sequencing to identify tomato miRNAs related to resistance against *P. infestans* as well as on the functional analysis of *P. infestans*-induced miRNAs in tomato, including miR169, miR398, miR482, miR6024 miR6026, miR6027^27^,^28^,^29^. MiR1918 is expressed in all tomato tissues, and it was initially thought to be involved in the regulation of fruit development and maturation^30^. Recently, a number of *P. infestans* miRNAs (pi-miRNAs) have been predicted using *P. infestans* EST data and all the known miRNAs from miRBase, and pi-miR1918 is among these predicted pi-miRNAs, and its level was found to change dramatically in *P. infestans*-infected *Solanum pimpinellifolium* leaves^31^. In addition, a recent silico study has shown that tomato miR1918 might bind to the AC1 gene coding the replication-related protein in Tomato leaf curl virus (ToLCV). This suggests that it might also target the AC1 gene and inhibit ToLCV replication^32^.

The present study aimed to understand the roles of miR1918 in tomato -*P. infestans* interaction by analyzing the expressions of miR1918 and its target genes in tomato and *P. infestans* during the tomato-*P. infestans* interaction. The result suggested that miR1918 enhanced the susceptibility of the tomato to *P. infestans* infection.

## Results

### Tomato and *P. infestans* transcripts targeted by miR1918

**Figure 1:**
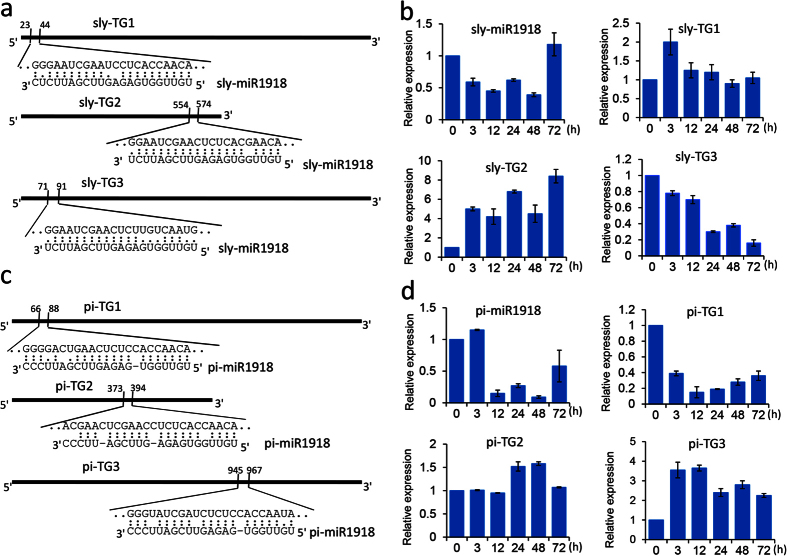
Target prediction of sly- and pi-miR1918 from tomato and *Phytophthora infestans* database and expression analyses of sly- and pi-miR1918 and their targets genes during tomato - *P. infestans* interaction. (**a**) Regions targeted by sly-miR1918 in tomato transcripts. (**b**) Expression profile of sly-miR1918 and its targets after *P. infestans* infection. sly-TG1, Os12g0283800 protein; sly-TG2, RING finger gene; sly-TG3, MATE efflux family gene. (**c**) Regions targeted by pi-miR1918 in *P. infestans* transcripts. (**d**) Expression profiles of pi-miR1918 and its targets in *P. infestans*. pi-TG1, Secreted RxLR effector peptide protein; pi-TG2, trafficking protein particle complex subunit; pi-TG3, Phosphoethanolamine N-methyltransferase.

To investigate the potential role of miR1918 during tomato-*P. infestans* interaction, we first predicted the target genes of sly- and pi-miR1918 through searching the tomato and *P. infestans* databases, respectively. Using psRNATarget and TargetAlign, which are bioinformatics tools for identifying mRNAs targeted by miRNA, we obtained a number of target genes for miR1918. 56 target genes of sly-miR1918 were predicted from tomato cDNA library by psRNATatget (maximum expectation ≤ 4) (Table S1). Three target genes which were maximum expectation ≤ 2.5, Os12g0283800-like gene (*sly-TG1*, Solyc10g045620.1.1), RING finger (*sly-TG2*, Solyc01g095820.2.1) and MATE efflux gene (*sly-TG3*, Solyc10g054110.1.1) were selected for next analysis. Little is known about the function of *sly-TG1,* but the functions of *sly-TG2* and *sly-TG3* have already been demonstrated. *Sly-TG2* is generally associated with ubiquitination implicated in plant defense against biotic and abiotic challenges^33^,^34^ and *sly-TG3* is an essential component of SA and ABA-dependent signaling for disease and abiotic stress resistance^35^,^36^. As for pi-miR1918, three target genes were predicted from the data of *P. infestans* mRNAs and 3′UTR sequences by TargetAlign. These were secreted RxLR effector peptide protein (*pi-TG1*, PITG_19655), trafficking protein particle complex subunit (*pi-TG2*, PITG_14806) and phosphoethanolamine N-methyltransferase (*pi-TG3*, PITG_15903) (Fig. 1a,c).

Quantitative real time RT-PCR (*q*RT-PCR) was used to examine the expression of sly-miR1918 and pi-miR1918 as well as their target genes in tomato and *P. infestans*, respectively after infection with *P. infestans*. The expression of sly-miR1918 was down-regulated except at 72 hpi (Fig. 1b). Its target gene *sly-TG1* was up-regulated during the first 3 hpi, whereas *sly-TG2* was up-regulated in the first 24 hpi. The expression of *sly-TG3* actually decreased at all time points following infection. As for pi-miR1918, its expression during the first 3 hpi was not significantly changed, but was decreased slightly after 3 hpi. The expression of *pi-TG3* increased at all time points following infection (Fig. 1d). In addition, the expression of secreted *pi-TG1* was negatively correlated with that of pi-miR1918 from during the first 3 hpi and from 24 to 48 hpi, whereas the expression of secreted *pi-TG2* was negatively correlated with that of pi-miR1918 from 24 to 72 hpi.

### Construction of artificial *P. infestans* miR1918 vector

**Figure 2:**
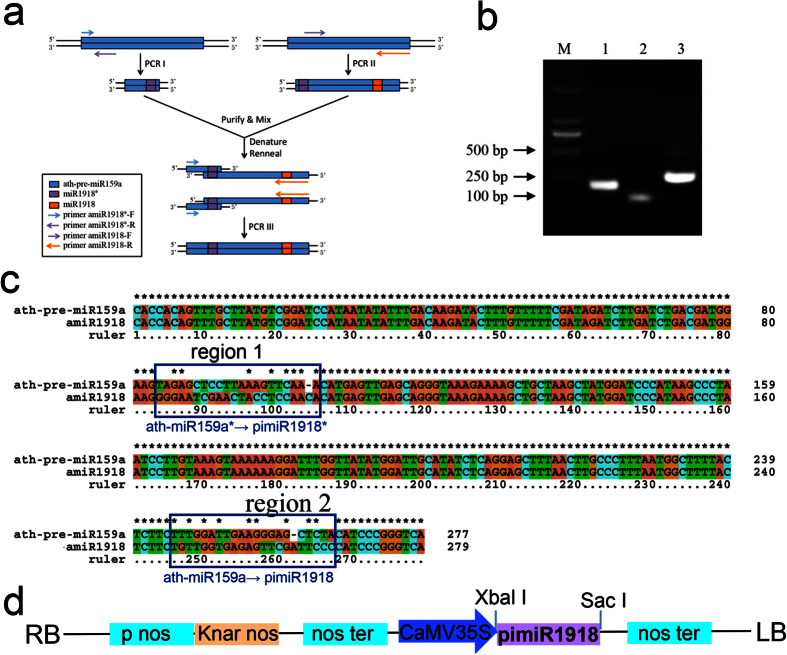
Construction of artificial miR1918 expression plasmid. (**a**) Strategy for introducing mutations by PCR. (**b**) PCR products from the different stages of the artificial pi-miR1918 generation. M, DL2000 DNA Marker; 1, 2 and 3, products from the first, second and third rounds of PCR, respectively. (**c**) Sequence alignment of ath-pre-miR159a and pi-amiR1918 performed with ClustalX. Boxes represent ath-miR159a and ath-miR159a* mutation in pi-miR1918 and pi-miR1918*. (**d**) Schematic representation of pBI121-pi-amiR1918 plasmid showing the sites for restriction enzymes.

As previous studies have shown that miR159 (ath-miR159) is expressed at high levels in *A. thaliana*, the hairpin structure of ath-pre-miR159a was chosen as the backbone for the design of amiRNA. The design of artificial *P. infestans* miR1918 (pi-amiR1918) is shown in Fig. 2a. Oligonucleotide-directed mutagenesis was used to replace ath-miR159a and ath-miR159a* with pi-miR1918 and pi-miR1918*, respectively. The last round of PCR was performed to amplify the sequence of pi-amiR1918. The products from these three rounds of PCR were detected using agarose gel electrophoresis. The lengths of the amplified fragments were consistent with the expected sizes (Fig. 2b). In addition, sequence alignment between ath-pre-miR159a and pi-amiR1918 showed that both sequences were conserved except for two regions of 21-nt, marked as region 1 and region 2 in Fig. 2c. Pi-miR1918 was derived from ath-miR159a* by a mutation within region 1 or from ath-miR159a by a mutation within region 2. The secondary structures of ath-pre-miR159a and pi-amiR1918 obtained with Mfold 3.5 were consistent each other (Fig. S1). Overall, the data suggested that pi-amiR1918 was successfully constructed by using ath-pre-miR159a as the backbone.

To further investigate the role of miR1918, a plasmid for the overexpression of pi-amiR1918 was constructed on the basis of the pBI121 vector. The DNA containing the pi-amiR1918 sequence was cloned into *Xbal* I-*Sac* I cut pBI121, placing it under the control of the Cauliflower mosaic virus (CaMV) 35S promoter (Fig. 2d). The pBI121-pi-amiR1918 construct obtained was sequenced to confirm the presence of pi-amiR1918 and the result of multiple sequence alignment between pBI121-pi-amiR1918 and pi-amiR1918 indicated that pBI121-pi-amiR1918 was successfully constructed (Fig. S2).

### Overexpression of amiR1918 by agrobacterium infiltration

**Figure 3:**
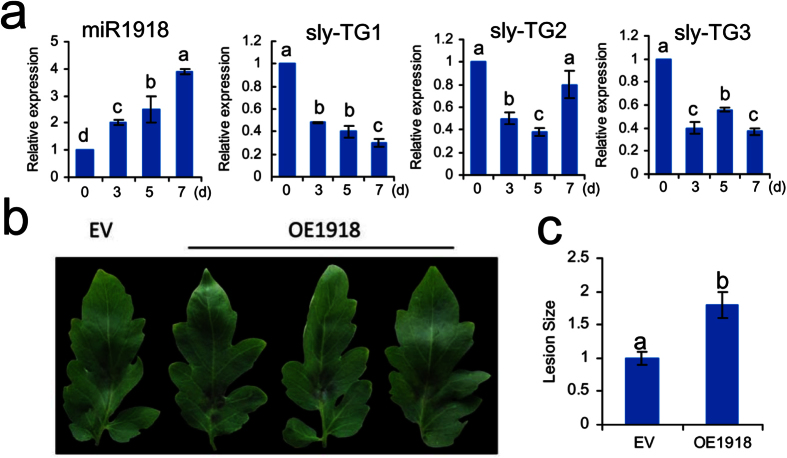
Transient overexpression of amiR1918 in tomato leaves. (**a**) Relative quantities of miR1918 and its targets in tomato leaves transiently over-expressing amiR1918. *sly-TG1*, Os12g0283800 gene; *sly-TG2*, RING finger gene; *sly-TG3*, MATE efflux family gene. (**b**) Disease symptoms of tomato leaves transiently overexpressing amiR1918 four days after inoculation with *P. infestans.* EV, transient overexpression empty vector; OE1918, transient overexpression of pBI121-pi-amiR1918. (**c**) Comparison of lesion sizes between EV and OE1918. Data are the means ± SEs of three independent experiments. Samples marked with various letters are significantly different (*p* < 0.05) according to Duncan’s Multiple Range Test.

The pBI121-pi-amiR1918 plasmid was mobilized into *Agrobacterium tumefaciens* strain GV3101 by the freeze-thaw method. As shown in Fig. S3, pBI121-pi-amiR1918 was successfully mobilized into GV3101 by PCR using the primers amiR1918*-F and amiR1918-R (Table S1). Introduction of *A. tumefaciens* harbouring pBI121-pi-ami1918 into tomato leaves resulted in significant up-regulation of miR1918 (sly-miR1918 and amiR1918) expression during the first seven days (Fig. 3a). The expression of miR1918 was also negatively correlated with the expression of the target genes. Three days after the transient overexpression of amiR1918, the leaves were infected with *P. infestans*. Resistance analysis showed that the extent of the disease was more severe and the lesions were significantly larger in *P. infestans-*infected leaves that transiently overexpressed amiR1918 (OE1918) than in *P. infestans-*infected leaves that did not overexpress amiR1918 (EV) (Fig. 3b,c). These results suggested that overexpression of amiR1918 may have weakened the resistance of tomato against *P. infestans.*

### Overexpression of amiR1918 in tomato enhances sensitivity to *P. infestans* infection

**Figure 4:**
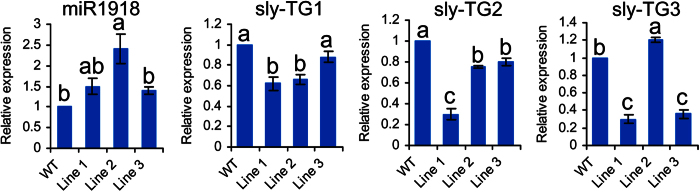
Quantitative real-time PCR analysis of the expression levels of miR1918 and its target genes in three selected transgenic lines of tomato.

To investigate whether miR1918 is involved in tomato-*P. infestans* interaction, pBI121-pi-amiR1918 was introduced into tomato. After callus induction and shoot regeneration, three representative positive transgenic lines were confirmed by selection on medium containing kanamycin. Four-week-old tomato seedlings (WT and transgenic lines) were transferred to pots filled with perlite/vermiculite (1:3, v/v) and grown for 2 weeks before they were subjected to *q*RT-PCR analysis to estimate the levels of miR1918 expression in the leaf tissues (Fig. 4). Compared to the wild types, all the transgenic lines expressed a higher level of amiR1918, with the levels of amiR1918 in Line 1, Line 2 and Line 3 being approximately 1.5 fold, 2.4 fold, and 1.4 fold the level of amiR1918 in WT, respectively.

**Figure 5:**
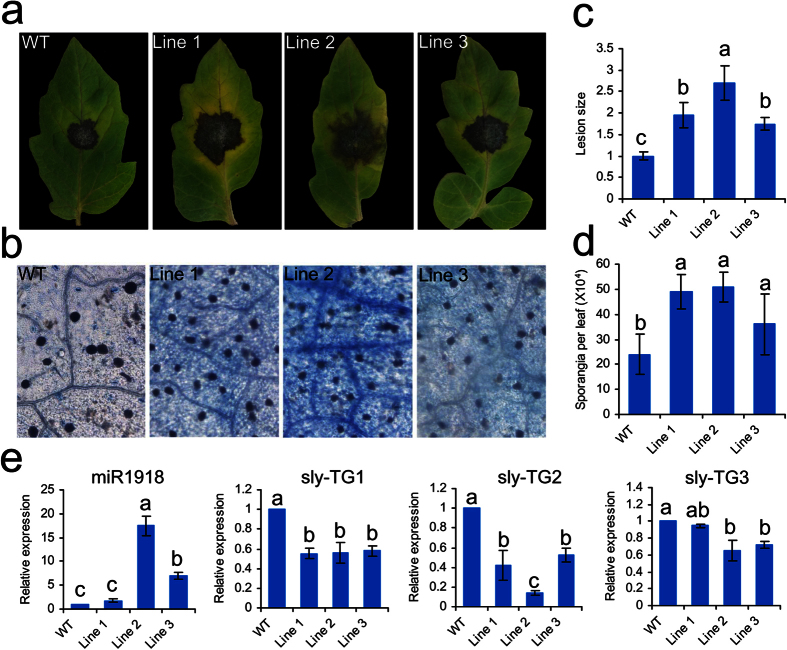
Effect of amiR1918 overexpression on the resistance of transgenic tomato to *Phytophthora infestans*. (**a**–**d**) Parameters showing the extent of the disease seven days after infection with *P. infestans.* (**a**) Disease symptoms; (**b**) number of necrotic cells (Scale bars:100 μm); (**c**) lesion size; (**d**) sporangia per leaf. (**e**) Expression patterns of miR1918 and its target genes in tomato leaves from WT and transgenic lines at 7 days after inoculation with *P. infestans*. Data are the means ±SEs of three independent experiments. Samples marked with various letters are significantly different at the p < 0.05 level according to Duncan’s Multiple Range Test.

The three transgenic and WT tomato plants were infected with *P. infestans*, and seven days after the infection, the physical appearance of the plants was assessed. The leaves of the transgenic plants showed necrotic lesions, whereas the leaves of WT plants exhibited fewer symptoms of the disease (Fig. 5a). Trypan blue staining also revealed increased sensitivity to the pathogen in the case of the transgenic plants, as shown by the presence of increased number of necrotic cells compared to WT (Fig. 5b). In addition, the transgenic plants also had larger lesion size and more sporangia per leaf than the WT plants (Fig. 5c,d). These results suggested that overexpression of amiR1918 enhanced the susceptibility of the plants to *P. infestans* infection.

### The identification of target genes by 5′ RACE

The expressions of the target genes also varied between transgenic lines and WT. In the case of *sly-TG1,* its level in Line 1 was approximately 1.5 fold that of WT, but did not really increase in Line 2. As for *sly-TG2,* its level in Line 1 was approximately 3.3 fold that of WT. In the case of *sly-TG3,* its levels in Line 1 and Line 3 were about 3 fold that of WT. Intriguingly, the expression level of amiR1918 was the highest in Line 2. Also in Line 2, the expressions of *sly-TG1* and *-TG2* were down-regulated, whereas the expression of *sly-TG3* was up-regulated. The expression pattern analysis showed there was no negative correlation between the expression of amiR1918 and the expression of *sly-TG1* and *sly-TG3* (Fig. 1b). In addition, we also used another reference control gene, α-tubulin to perform the qRT-PCR analysis in tomato. The results indicated there was negative correlation between expression of miR1918 and the expression of *sly-TG2* and only *sly-TG2* expression was down-regulated in all transgenic lines (Fig. S4).

The expression of miR1918 and its target genes (*sly-TG1*, *sly-TG2* and *sly-TG3*) in WT and three transgenic lines were also detected by *q*RT-PCR seven days after infection with *P. infestans*. The expression of miR1918 was significantly up-regulated in Line 2 and Line 3 (*p* < 0.05), but not in Line 1. The expressions of *sly-TG1* and *sly-TG2* were down-regulated in all three transgenic lines, while the expression of *sly-TG3* was significantly down-regulated in Line 2 and Line 3 (Fig. 5e). However, the most significant changes in the expression levels of miR1918 and its target genes were detected in Line 2.

To identify the target genes of miR1918 in tomato, we used the 5′ RACE to determine the cleavage site. Total RNA was extracted from overexpression miR1918 tomato (Line 2); 5′ RNA adapter was ligated using T4 RNA ligase; the ligated mRNA was reversed using Oligo dT. After PCR and Sanger sequence, we only found that 3 out of 10 cloned sequence had the same cleavage site of *sly-TG2* between 10^th^ nucleotide and 11^th^ nucleotide (Fig. S5). This suggested that only *sly-TG2* was the target genes of miR1918 in tomato.

## Discussion

As a novel type of regulatory factor, plant miRNAs play an important role in plant-pathogen interaction^37^. For instance, our previous work has shown that the miR396 family miRNAs target growth-regulating factor (GRF) genes. Tobacco plants overexpressing *Sp*-miR396a-5p show increased susceptibility to *P. nicotianae* infection^38^. Although plant miRNAs have been extensively studied, few miRNAs have been discovered in microbes. Computational analysis has identified 78 mRNAs as potential target transcripts of 14 milRNAs from *M. anisopliae*^39^ A number of miRNAs-like RNAs have been identified in the plant fungal pathogen *Sclerotinia sclerotiorum* by high-throughput sequencing^11^. In our previous study, some miRNAs in *P. infestans,* including miR1918, were predicted from the *P. infestans* EST data and all the known miRNAs from miRBase^27^. *P. infestans* and tomato are two unrelated organisms, but both contain the conserved sequence of miR1918, and therefore studying the function of miR1918 would be of great significance.

RNA silencing is considered to be an innate immune response in plant and it is important that a pathogen evades this line of defense in order to establish successful infection. Small RNA silencing that targets endogenous mRNAs may have a distinct biogenesis and involves miRNAs or siRNAs. During plant-microbe interaction, plant miRNAs may regulate their own effector genes or transposable elements (TEs)^12^, and RNA silencing further affects the resistance of a plant to pathogen^29^. Besides, sRNAs have been reported to move horizontally between plant and pathogen. Plant miRNAs can be transferred to viruses, and in return pathogen-derived sRNAs can bind to host transcripts when the plant interacts with microbes, including virus and fungus^12^,^16^,^17^,^40^. Overall, a plant can recognize and develop various means to defend itself against the attacks by pathogens, and thus the pathogens need to have strategies that would facilitate their infection and create a suitable environment to grow and reproduce inside the host plant. A negative correlation was observed between the expression of pi-miR1918 and some of its targets in tomato infected with *P. infestans* (Fig. 1b). Moreover, sly-miR1918 was detected in tomato leaves inoculated with *P. infestans* and its expression level was down-regulated at all time points except at 72 h following the inoculation. Its target genes, *sly-TG1*and *sly-TG2* were up-regulated following the inoculation, although at different time points (Fig. 1a). These results suggested that pi- and sly-miR1918 could silence their respective targets.

Using 5′-RACE, *sly-TG2* was identified as the target genes of miR1918 in tomato. *Sly-TG2* (RING finger gene) belongs to a large family of zinc finger-coding genes that play important roles in the regulation of growth and development, hormone signaling, and responses to biotic and abiotic stresses in plants. RING finger proteins, which are generally associated with ubiquitination, are implicated in plant defense against biotic and abiotic factors^33^,^34^. For example, the expression of a RING finger protein-coding gene from the Chinese wild grapevine *Vitis pseudoreticulata* (designated VpRFP1) has been shown to increase rapidly in mildew-resistant *V. pseudoreticulata* plants upon inoculation with grapevine powdery mildew *Uncinula necator*^41^. Overexpression of VpRFP1 in *Arabidopsis* plants has been found to enhance resistance to *Arabidopsis* powdery mildew *Golovinomyces cichoracearum* and a virulent bacterial pathogen *Pseudomonas syringae* pv. *tomato* DC3000. Another *V. pseudoreticulata* RING finger protein, EIRP1, can activate plant defense responses by inducing proteolysis of the VpWRKY11 transcription factor^42^. Furthermore, the expression of a novel E3 ubiquitin ligase RING1 gene (CaRING1) from pepper (*Capsicum annuum*) was found to be induced upon infection with avirulent *Xanthomonas campestris* pv *vesicatoria*, and overexpression of CaRING1 in *Arabidopsis* can confer enhanced resistance to infection by hemibiotrophic *Pseudomonas syringae* pv *tomato* and biotrophic *Hyaloperonospora arabidopsidis*^43^. On the basis of these results, genes coding for RING finger proteins could be key regulators of plant immunity. Our results showed that amiR1918 was negatively correlated with the expression of RING finger-coding genes and that there were more disease symptoms and larger lesion size in the transgenic tomato plants (Fig. 4). This suggested that amiR1918 might be involved in silencing genes coding for RING fingers, a process that would eventually result in the tomato having enhanced susceptibility to *P. infestans* infection.

The predicted targets of pi-miR1918 in *P. infestans* might play important roles in tomato-*P. infestans* interaction. For example, pi-TG3 (which codes for phospoethanolamine N-methyltransferase) is related to glycerophospholipid metabolism. It participates in the synthesis of stress-associated second messenger such as phosphatidic acid. Phosphatidic acid can regulate various signal pathways that might be involved in the susceptibility of tomato to *P. infestans* infection. The RxLR effector gene, *pi-TG1* is a target gene of pi-miR1918 in *P. infestans*. RxLR effector protein mediates its own entry into host cells after being secreted from the pathogens^44^. One previous study has shown that an RxLR effector from *P. infestans* can prevent the re-location of two plant NAC transcription factors to promote disease progression^45^. Another *P. infestans* RxLR effector interacts with host MAPKKK to suppress plant immune signaling^44^. In addition, RxLR effectors also suppress RNA silencing in plants, leading to enhanced susceptibility to *Phytophthora*. Two RxLR-like effectors, PSR1 and RSR2 are found in the oomycete plant pathogen *P. sojae*^46^. PSR2 is a member of the conserved RxLR effector family that is widely distributed in *Phytophthora* spp and it may contribute to the virulence of the fungus by suppressing host RNA silencing processes in various plants^47^. On the other hand, PSR1 can target PINP1 (a previously unidentified component of RNA silencing that regulates distinct classes of small RNAs in plants) in order to promote infection^48^.

In conclusion, an artificial pi-miR1918 was generated by PCR using the precursor of *A. thaliana* pre-miR159a as the backbone, and tomato plants that overexpressed this artificial pi-miR1918 displayed more serious disease symptoms than WT plants after infection with *P. infestans.* A few target genes of miR1918 were predicted for tomato and their expressions were found to be negatively correlated with the expression of miR1918. These results suggested that miR1918 might be involved in the silencing of target genes, thereby enhancing the susceptibility of tomato to *P. infestans* infection.

## Materials and Methods

### MiR1918 target prediction from tomato and *P. infestans* database

The identification of mRNAs targeted by sly- and pi-miR1918 was performed using psRNATarget (http://plantgrn.noble.org/psRNATarget/) and TargetAlign (http://www.leonxie.com/targetAlign.php), respectively. Tomato transcripts (cDNA library, version 2.4) and sly-miR1918 sequence were used as data sets. The parameters were as follow: (i) Maximun expectation: 3; (ii) Length for complementarity scoring (hspsize): 20; (iii) Length for complementarity scoring (hspsize): 200; (iv) Target accessibility - allowed maximum energy to unpair the target site (UPE): 25; (v) Flanking regions around the target site for target accessibility analysis: 13 bp downstream and 17 bp upstream; (vi) Range of central mismatch leading to translational inhibition: 9–11nt. In addition, the target genes of pi-miR1918 were predicted from the *P. infestans* database (http://www.broadinstitute.org/) using TargetAlign. The sequence of pi-miR1918 was obtained from our previous work^23^.

### Collection of tomato and *P. infestans* specimens during their interaction

The tomato plant *S. lycopersicum Zaofen No.2* was chosen as the host plant. It was grown in a greenhouse under 16 h light at a temperature range of 22–28 °C. *P. infestans* strain P12103 used in the experiments was provided by Prof. Shan from Northwest A&F University of China. The specimen had been collected from Pingliang City, Zhuanglang County, Gansu Province, China, in 2009. The treated tomato plants (Ts, 5-6-leaf stage) were inoculated with a suspension of *P. infestans* spores (10^6^ zoospores/mL; 5 individuals each) before being placed in a 100% relative humidity room or chamber in the dark to ensure spore germination at 20 ± 1 °C. The controls (Cs, 5 individuals) were treated with the same volume of sterile water and grown under the same conditions. Leaf samples were collected from each plant immediately after inoculation (0 hpi) and at 3, 12, 24, 36, 48 and 72 hpi, and the leaves from Ts and Cs plants were pooled.

*P. infestans* P12103 was maintained on oat medium that had been covered with a layer of cellophane inside a 9-cm culture dish. The dish was kept at 20 °C in the dark. After 10 days, the cellophane, which was filled with *P. infestans,* was transferred to a new culture dish. Tomato leaves were used to cover the upper and lower sides of this cellophane as described previously^31^. *P. infestans s*amples were collected from the cellophane at 0, 3, 12, 24, 36, 48 and 72 hpi. All samples were quickly frozen in liquid nitrogen and stored −80 °C for storage until DNA and RNA isolation.

### Construction of amiR1918 vector

AmiR1918 was generated by PCR, which involved amplification and mutagenesis using the precursor of ath-pre-miR159a as the backbone^21^,^49^. The sequence of ath-pre-miR159a was cloned by PCR using the primers, ath-pre-miR159-F and ath-pre-miR159-R (Table S1). The PCR fragment was cloned into pMD-19T vector (TaKaRa) according to the manufacturer’s instruction.

To construct pi-amiR1918, four primers were designed: amiR1918-F, amiR1918-R, amiR1918*-F and amiR1918*-R (Table S1). Three rounds of PCR were performed to amplify pi-amiR1918. In the first round of PCR, pMD-19T-ath-pre-miR159a, amiR1918-F and amiR1918-R were used to amplify a fragment containing miR1918. In the second round of PCR, pMD-19T-ath-pre-miR159a, amiR1918^*^-F and amiR1918^*^-R were used to amplify a fragment containing miR1918^*^. In the third round of PCR, the PCR products of the first and second rounds were used as the template along with amiR1918^*^-F and amiR1918-R. The fragment from the third round of PCR was cloned to pMD-19T and its sequence was checked by DNA sequencing.

### Plasmid construction and transient overexpression in tomato

The DNA fragment obtained from the third round of PCR was digested with *Sac*I and *Xba*I and then cloned into *Sac*I-*Xba*I digested pBI121 to generate the recombinant plasmid pBI121-pi-ami1918, in which pi-amiR1918 was placed under the control of the strong constitutive CaMV35S promoter. This recombinant plasmid was transformed into *A. tumefaciens* strain GV3101 by freeze-thaw method.

A sample from *A. tumefaciens* culture was centrifuged at 4,000 × g for 10 min and the supernatant was discarded. The pellet was resuspended in infiltration medium (10 mM MES, 10 mM MgCl2 and 20 μM acetosyringone) (OD600 = 0.1). *Agrobacterium tumefaciens* was introduced into tomato leaves by infiltration. *Agrobacterium tumefaciens* with empty vector was used as a control. After 3 days, the infiltrated leaf regions were inoculated with 20 μl of *P. infestans* (10^6^ zoospores/mL) per infiltrated leaf region. These regions were collected for RNA extraction at the indicated times (0, 2 and 4 d). In addition, the sizes of the lesions were calculated from the image obtained by ImageJ^17^.

### Generation of pi-amR1918 transgenic tomato lines and ***P. infestans*** resistance analysis

Tomato cotyledon were infected with *A. tumefaciens* strain GV3101 containing the modified plasmid pBI121-pi-ami1918 via *Agrobacterium*-mediated leaf disk method. Putative transgenic plants were selected on Murashige and Skoog (MS) agar medium containing 50 mg l^−1^ kanamycin.

Four-week-old tomato seedlings (WT and transgenic lines) were transferred to pots filled with perlite/vermiculite (1:3, v/v) and grown for 2 weeks. The expression levels of mR1918 and target genes were examined by *q*RT-PCR. The plants (WT and transgenic) were each infected with *P. infestans* by inoculating with a suspension of *P. infestans* spores. After 4 days, the leaves were collected for RNA extraction to examine the expression levels of pi-amiR1918 and target genes. After 7 days, the relative lesion size, sporangia per leaf, and detection of dead cells were determined by ImageJ, direct counting and trypan Blue staining^38^, respectively.

### RNA isolation and reverse transcription

Total RNA was extracted from tomato using RNAiso Plus (TaKaRa) and the corresponding cDNAs were synthesized with SYBR^®^ PrimeScriptTM miRNA RT-PCR Kit (TaKaRa) and PrimeScriptTM RT Master Mix (TaKaRa) according to the manufacturers’ instructions.

### qRT-PCR analysis

Quantitative real-time PCR (*q*RT-PCR) was used to analyze the expression levels of miR1918 and its target genes. The specific forward primers for each miRNA gene were designed according to a previously described method^50^, and *q*RT-PCR was performed with the SYBR^®^ PrimeScriptTM miRNA RT-PCR kit (TaKaRa) according the manufacturer’s protocol using the ABI 7300 Fast Real-time PCR machine (Applied Biosystems, Foster City, CA, USA). The primers for the target genes were designed following the manufacturer’s instructions as described in the SYBR^®^ Premix Ex TaqTM II kit (TaKaRa). The presence of miRNA-mediated cleavage of target genes at a site located between the Forward and Reverse primers was ensured. Actin andα-tubulin were used as reference control gene for the miRNA and target gene *q*RT-PCR analysis. The sequence information of all primers is provided in Table S2. Mixed samples were used in *q*RT-PCR analysis, and three biologically independent replicates were analyzed for each sample.

### 5′-Rapid amplification of cDNA ending (5′-RACE) analysis

5′-RACE was used to determine the cleavage site of miR1918 on target genes in tomato. The GeneRacer TM (Invitrogen) kit was used to perform the 5′-RACE.according the manufacturer’s protocol. Total RNA was extracted from overexpression miR1918 tomato (Line 2), 5′ RNA adapter was ligated using T4 RNA ligase and the ligated mRNA was reversed using Oligo dT. The sequence information was provided in Table S2.

### Statistical analysis

All statistical analysis of data was performed with SPSS, and all data were expressed as the means ± SEs from three independent experiments. The Duncan’s multiple range test (*p* < 0.05) was selected where was appropriate.

## Additional Information

**How to cite this article**: Luan, Y. *et al*. MiR1918 enhanced tomato sensitivity to *Phytophthora infestans* infection. *Sci. Rep.* **6**, 35858; doi: 10.1038/srep35858 (2016).

## Supplementary Material

Supplementary Information
