# Supplementary material for: MiR1918 enhances tomato sensitivity to Phytophthora infestans infection
Source: Sci Rep. 2016 Oct 25;6:35858. doi: 10.1038/srep35858 (PMC5078808; doi:10.1038/srep35858)
Supplement: Supplementary Information [file srep35858-s1.doc]

MiR1918 enhances tomato sensitivity to *Phytophthora infestans* infection

Yushi Luan^1^. Jun Cui^1^. Weichen Wang^1^. Jun Meng^2*^

^1^ School of Life science and Biotechnology, Dalian University of Technology, Dalian 116024, China

^2^ School of Computer Science and Technology, Dalian University of Technology, Dalian 116024, China

^*^ Correspondence author

Tel: +86 411 84706003

Fax: +86 411 84706365

E-mail address: [mengjun@dlut.edu.cn](mailto:mengjun@dlut.edu.cn)


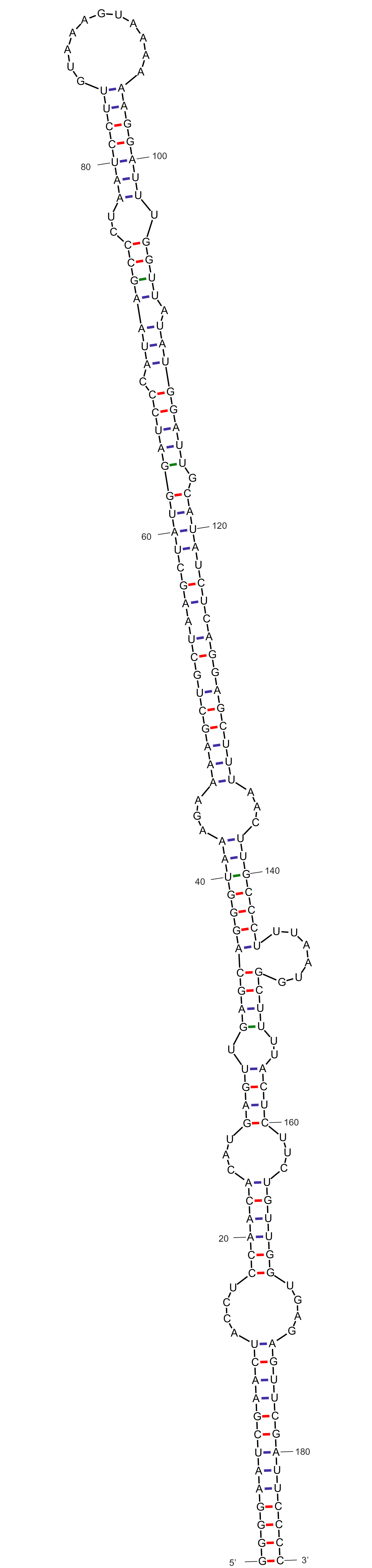


**ath-miR159a/ath-miR159a***

**pi-miR1918/pi-miR1918***

**Figure S1** Secondary structures of ath-pre-miR159a and pi-amiR1918 as depicted by Mfold 3.5.
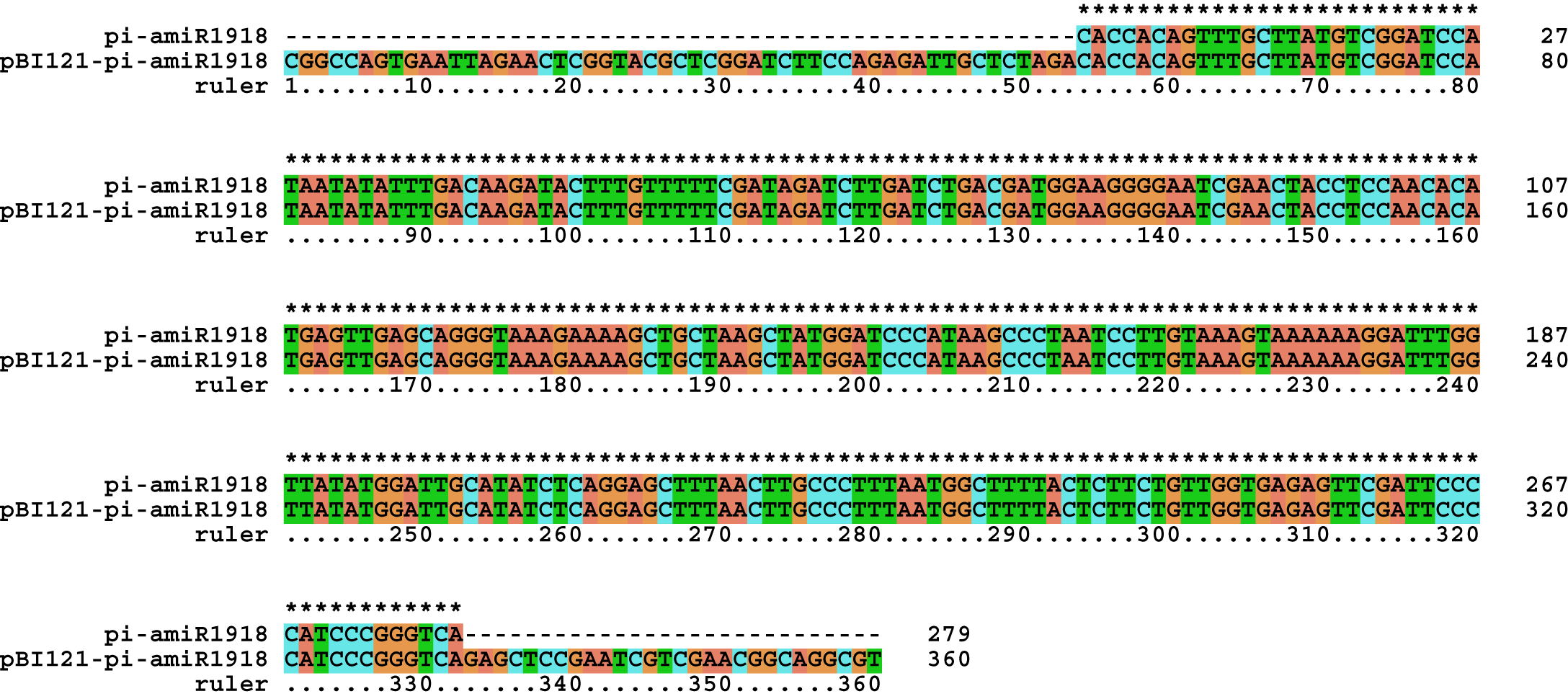


**Figure S2** Sequence alignment between pBI121-pi-amiR1918 and pi-amiR1918.


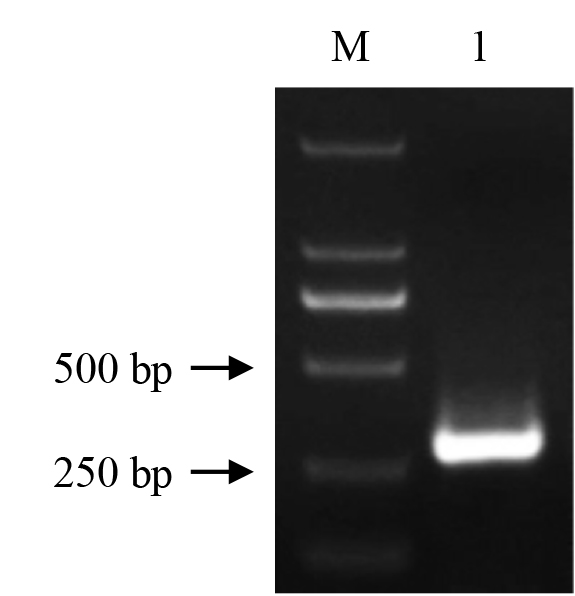


**Figure S3** Detection of artificial pi-miR1918 from GV3101 with pBI121-pi-amiR1918.


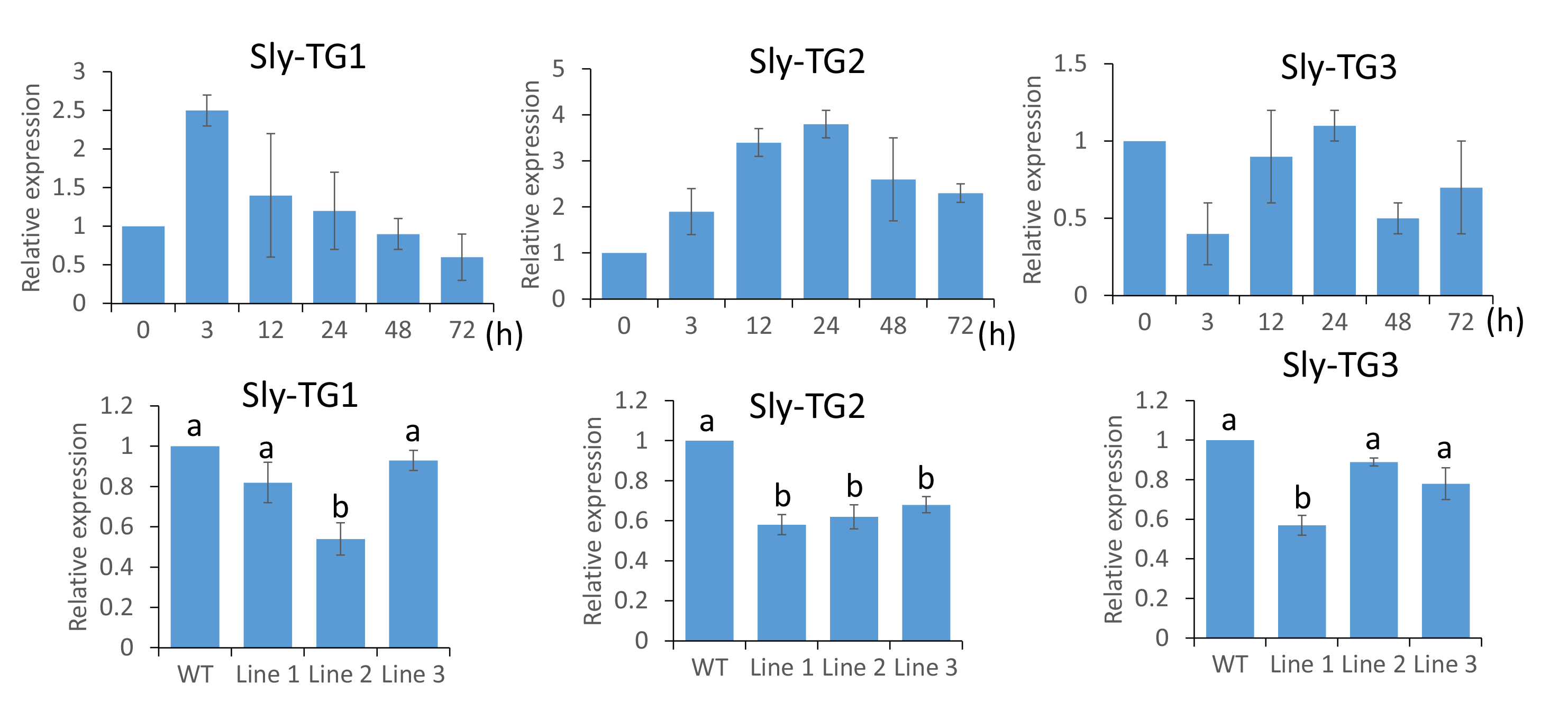


a

b

**Figure S4** The qRT-PCR analysis using α-tubulin as reference control gene. (a) The expression analyses of sly- miR1918 and their targets genes during tomato - *P. infestans* interaction. (b) Quantitative real-time PCR analysis of the expression levels of target genes of miR1918 in three selected transgenic lines of tomato


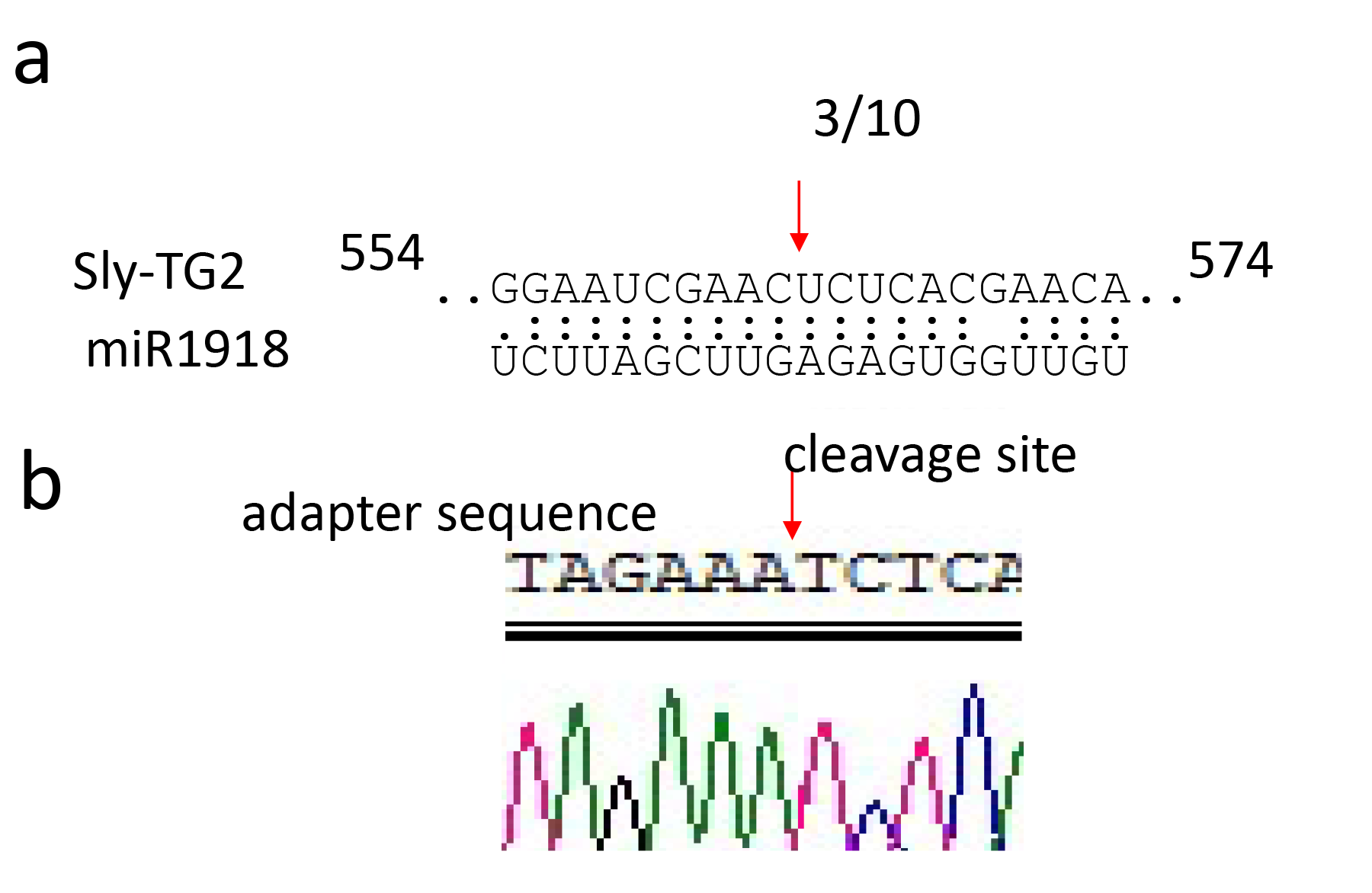


**Figure S5** The results of 5’-RACE. (a) The cleavage site of miR1918 on *sly-TG2*. (b) The result of sanger sequence. The red arrows indicated the cleavage site.

**Table S2 the predicted genes of miR1918 in tomato**

| **miRNA_Acc.** | **Target_Acc.** | **Expectation** | **UPE** | **Target_start** | **Target_end** |
| --- | --- | --- | --- | --- | --- |
| miR1918 | Solyc10g045620.1.1 | 1.5 | 17.386 | 23 | 44 |
| miR1918 | Solyc01g095820.2.1 | 1.5 | 21.676 | 554 | 574 |
| miR1918 | Solyc10g054110.1.1 | 2.5 | 15.325 | 71 | 91 |
| miR1918 | Solyc06g054310.1.1 | 4 | 21.38 | 398 | 419 |
| miR1918 | Solyc06g076090.2.1 | 3.5 | 17.573 | 1367 | 1388 |
| miR1918 | Solyc01g007190.2.1 | 4 | 11.77 | 197 | 217 |
| miR1918 | Solyc01g105180.2.1 | 3 | 17.409 | 1433 | 1454 |
| miR1918 | Solyc01g028840.1.1 | 3.5 | 8.449 | 232 | 252 |
| miR1918 | Solyc02g063460.1.1 | 3.5 | 5.71 | 150 | 169 |
| miR1918 | Solyc04g040160.2.1 | 3.5 | 9.755 | 204 | 224 |
| miR1918 | Solyc01g090820.1.1 | 3.5 | 15.619 | 563 | 582 |
| miR1918 | Solyc03g123760.2.1 | 3.5 | 15.968 | 923 | 942 |
| miR1918 | Solyc06g074650.2.1 | 4 | 16.158 | 2701 | 2722 |
| miR1918 | Solyc10g055470.1.1 | 4 | 16.474 | 857 | 877 |
| miR1918 | Solyc07g008080.2.1 | 4 | 20.416 | 1411 | 1431 |
| miR1918 | Solyc04g056480.1.1 | 4 | 20.991 | 91 | 111 |
| miR1918 | Solyc02g091330.2.1 | 3.5 | 19.147 | 311 | 331 |
| miR1918 | Solyc01g107000.1.1 | 3 | 10.961 | 582 | 602 |
| miR1918 | Solyc03g043900.1.1 | 4 | 20.131 | 120 | 141 |
| miR1918 | Solyc10g047030.1.1 | 4 | 19.447 | 544 | 565 |
| miR1918 | Solyc03g006920.2.1 | 4 | 9.94 | 2218 | 2237 |
| miR1918 | Solyc07g022910.2.1 | 4 | 13.526 | 1509 | 1530 |
| miR1918 | Solyc03g123520.2.1 | 4 | 21.577 | 2102 | 2123 |
| miR1918 | Solyc04g076200.2.1 | 4 | 14.287 | 228 | 247 |
| miR1918 | Solyc08g077840.2.1 | 4 | 24.225 | 645 | 667 |
| miR1918 | Solyc11g018500.1.1 | 4 | 14.656 | 821 | 841 |
| miR1918 | Solyc04g025380.1.1 | 4 | 5.386 | 136 | 156 |
| miR1918 | Solyc11g017060.1.1 | 4 | 21.266 | 191 | 212 |
| miR1918 | Solyc12g017880.1.1 | 3.5 | 12.686 | 128 | 148 |
| miR1918 | Solyc02g093460.2.1 | 4 | 14.49 | 1590 | 1610 |
| miR1918 | Solyc01g106260.2.1 | 4 | 18.383 | 1037 | 1057 |
| miR1918 | Solyc01g106210.2.1 | 4 | 19.091 | 1089 | 1109 |
| miR1918 | Solyc07g040940.2.1 | 4 | 15.969 | 1071 | 1091 |
| miR1918 | Solyc08g067610.2.1 | 4 | 17.967 | 2411 | 2430 |
| miR1918 | Solyc06g050510.2.1 | 3.5 | 13.737 | 978 | 998 |
| miR1918 | Solyc10g017570.2.1 | 4 | 17.479 | 370 | 390 |
| miR1918 | Solyc06g007960.2.1 | 4 | 10.145 | 39 | 60 |
| miR1918 | Solyc08g013830.1.1 | 3.5 | 17.281 | 1211 | 1230 |
| miR1918 | Solyc01g106540.2.1 | 4 | 17.96 | 1427 | 1448 |
| miR1918 | Solyc10g006720.2.1 | 4 | 11.883 | 2380 | 2400 |
| miR1918 | Solyc05g005070.2.1 | 4 | 18.354 | 195 | 214 |
| miR1918 | Solyc08g060930.2.1 | 3.5 | 14.593 | 195 | 214 |
| miR1918 | Solyc03g097070.2.1 | 4 | 13.798 | 1387 | 1406 |
| miR1918 | Solyc03g080010.2.1 | 4 | 16.183 | 248 | 268 |
| miR1918 | Solyc08g078220.2.1 | 3.5 | 14.602 | 2006 | 2025 |
| miR1918 | Solyc08g078230.2.1 | 3.5 | 14.602 | 276 | 295 |
| miR1918 | Solyc08g081640.2.1 | 3.5 | 14.636 | 4601 | 4620 |
| miR1918 | Solyc09g060080.2.1 | 4 | 21.669 | 5925 | 5944 |
| miR1918 | Solyc10g048140.1.1 | 4 | 12.887 | 715 | 735 |
| miR1918 | Solyc01g079970.2.1 | 4 | 14.892 | 1174 | 1194 |
| miR1918 | Solyc01g079980.2.1 | 4 | 15.059 | 1173 | 1193 |
| miR1918 | Solyc06g053490.2.1 | 4 | 14.308 | 855 | 875 |
| miR1918 | Solyc01g079960.2.1 | 4 | 16.045 | 1165 | 1185 |
| miR1918 | Solyc02g089720.1.1 | 4 | 18.001 | 1846 | 1866 |
| miR1918 | Solyc06g075430.1.1 | 4 | 11.342 | 169 | 188 |
| miR1918 | Solyc03g034240.2.1 | 4 | 11.51 | 799 | 818 |

**Table S2 Primers used in PCR in this study**

| **Primer** | **Sequence (5’-3’)** |
| --- | --- |
| pi-miR1918 | TGTTGGTGAGAGTTCGATTCCC |
| sly-miR1918-F | TGTTGGTGAGAGTTCGATTCTC |
| pi-actin-F | GTTCCTGAGTTTTTGCTCCATC |
| pi-actin-R | GCAGACCCTTTGCTACTACCTT |
| sly-actin-F | TGTGTTGGACTCTGGTGATGGTGT |
| sly-actin-R | ATCCAAACGAAGAATGGCATGCGG |
| pi-TG1-F | ATCTGAATGGCGGTGTACTG |
| pi-TG1-R | CCTCTCTTCATCGTTCTCCTTG |
| pi-TG2-F | AGAACGAGGACGAGTACATGA |
| pi-TG2-R | CAGTCCAATTGCCCGAGAT |
| pi-TG3-F | TGAGAAACTCAACCTCCAACC |
| pi-TG3-R | AGCGCGGTGAACCATATT |
| sly-TG1-F | GGAATCGAATCCTCACCAACA |
| sly-TG1-R | GAGCTTAGTTTCTGCATGGGA |
| sly-TG2-F | CAGGTGGAAGGAATCGAACT |
| sly-TG2-R | GGACATGAGAAATCATAGCAACTC |
| sly-TG3-F | CCCAATACGGAATCGAACTCTT |
| sly-TG3-R | AGCTGAGCAAGCAGTGATTAT |
| amiR1918-F | GAAGGGGGAATCGAACTACCTCCAACACATGAGTTGAGCAGGGTAAA |
| amiR1918-R | CGGAGCTCTGACCCGGGATG***GGGAATCGAACTCTCACCAACA***GAAGAGTAAAAGCCATTAAA |
| amiR1918*-F | GCTCTAGACACCACAGTTTGCTTATGTCGGATCC |
| amiR1918*-R | TCATG***TGTTGGAGGTAGTTCGATTCCC***CCTTCCATCGTCAGATCAAG |
| ath-pre-miR159-F | CACCACAGTTTGCTTATGTCGGATCC-3 |
| ath-pre-miR159-R | TGACCCGGGATGTAGAGCTCCCTTCAATCC |
| α-tubulin-F | TGGTCGGAATGGGACAGAAG |
| α-tubulin-R | CTCAGTCAGGAGAACAGGGT |
| 5’ RNA adapter | CGACUGGAGCACGAGGACACUGACAUGGACUGAAGGAGUAGAAA |
| Oligo dT | GCTGTCAACGATACGCTACGTAACGGCATGACAGTG(T)_18_ |
| 5’ Primer | CGACTGGAGCACGAGGACACTGA |
| Gene specific primer | GGTTAGTTATATGAACTTCC |
